# The Increase of Sexual Debility in Males

**Published:** 1894-11

**Authors:** James Orr

**Affiliations:** Terrell, Texas


### TEXAS MEDICAL JOURNAL.

ESTABLISHED JULY, 1885.

PUBLISHED MONTHLY.—SUBSCRIPTION $2.00 A YEAR.

VOL. X. AUSTIN, NOVEMBER, 1894. NO. ~~4~~ 5

### Original Contributions.

For Texas Medical Journal.

### THE INCREASE OF SEXUAL DEBILITY IN MALES.

BY JAMES ORR, M. D., TERRELL, TEXAS.

[A paper read before the Terrell Medical Society, October 1, 1894.]

I BELIEVE it was Record who said his conception of Hades was a physician's office filled with importuning patients afflicted with gonorrhœal ailments. If such was his idea, I am sure contracted meatuses, strictures, gleets, and chronic bladder and kidney derangements, contributed but a small percentage to his woes when compared with inpotency, sterility, and kindred ailments, for I can imagine no greater supplicant than he whose sexual powers have already failed, or are in a constant state of impending bankruptcy.

Quacks and patent medicine vendors have been the greatest beneficiaries from this class of patients, many of whom, unfortunately, hesitate long ere making their conditions known to their usual medical attendants, hence large sums of money are annually thrown away in this rich field for humbuggery, where the harvest has been abundant, if the numerous and often costly advertisements of "Lost Manhood Restored," "Private Consultation Free," "Grand Turk's Restorative," "Dr. Blank's Invigorator," etc., are to be taken as an indication. This class of advertisements is on the increase, and I take it to be the experience of every regular practitioner that the number of men seeking legitimate medical aid for these diseases has greatly augmented within the past few years.

The importance of this question becomes apparent when considered in connection with the figures of a recent writer, who asserts that one-fourth of all men in the higher walks of life are sterile, and that forty per cent. of them, at the age of thirty-five, are either totally impotent, or suffer from sexual weakness, neurasthenia, or other form of sexual debility or insufficiency.

The Bibliotheca of this subject is limited, but few special books having been written upon it, while its magnitude and importance seem to have escaped the attention of "standard authors," as our medical and surgical encyclopedias devote but little space to its consideration, some contenting themselves with mentioning it only in connection with other diseases or ailments of which it is a symptom or concomitant. The excellent little work of Prof. S. W. Gross, on this subject, met a long felt want. In addition to this, a few other good books have been given to the profession. These, however, confine themselves mostly to treatment in connection with which their causative influences are considered only so far as it affects therapeutics, hence we find no moral prophylaxis mentioned. No danger signal waving across the pathway of our fellow men, who, yielding to the demands of business, the dictates of fashion and folly or the allurements of vice and licentiousness are implanting the seeds of physical degradation and moral decay in their limited offspring.

In considering the causes of these conditions, early medical writers confined themselves to the idea of sexual excesses or venereal diseases in the person afflicted, in which views Dr. Gross largely concurs. In fact, he gives stricture of the urethra as the prime factor in causing these ailments, nearly always finding it in victims of the habit of masturbation. So far, I have not read anything on the subject of microbes as a cause of sexual weakness, but I would not be surprised at any time to learn that some enterprising foreign investigator had discovered its pathogenic microbe, to which his unpronounceable name would be attached as a handle, that thereafter the little germ might go meandering down the corridors of time with its founder's name tied to it like a tin can to a dog's tail. But until some bacteriologist comes to our rescue, we common every-day practitioners must content ourselves with observing conditions, studying facts as we see them, or treating results.

There can be no question of the correctness of Dr. Gross' position in regard to some of the causes of sexual weakness, but I do not think he goes far enough, as anything causing deep-seated and long-continued urethral congestion will effect the sexual powers. In this way, congestion, inflammation and hypertrophy of the prostate, produces sexual neurasthenia, or other form of weakness, hence, in ivestigating these troubles the prostate and deep urethra must never be overlooked. A knowledge of these facts has recently directed the attention of medical men to "bicycle riding" as a potent factor in the production of congestion and hypertrophy of the prostate, with tissue changes in adjacent organs, for it places the entire weight of the body on a circumscribed area in the region of the prostate and deep seated urethra, where, if long continued, congestion, engorgement and chronic inflammation will inevitably ensue. In addition to this, the attitude of the "cyclist" is such as to place the utmost stretch upon the lower part of the spinal cord, thus producing irritation in the very region from which the nerves are distributed to the organs of generation. If these premises are correct, bicycle riding may be classed with the causes of the increase of sexual weaknesses among males.

Every observant physician must have noted the increase in the number of cases of nervous diseases among his patients, my own experience being that this type predominates, and the experience of other members of the profession with whom I have discussed it does not differ from mine. The cause of this increase of nervous derangements is to be found in weakened nervous systems due to habits and customs of our latter day civilization. We are developing our minds or filling our purses at the expense of our bodies; exhausting our energies, we perform our work by overdraft upon the future, the ultimate result of which is physical bankruptcy, and he who thus draws upon nature's reserve with the same reckless extravagance that the purse proud Baron draws upon his bank of gold is not the only sufferer from his folly, for his offspring yet unborn will bear the fruits of transmitted weaknesses. Again, our system of education is often one of overwork,—a species of cramming,—by which the mental and physical abilities of children are overtaxed, many pupils, particularly bright girls, being able to trace the origin of grave nervous diseases to this very cause. In all cases of overwork and mental strain, the nervous system is first to give way, the most delicate and complicate parts being the greatest sufferers, chief among which we find the organs of reproduction.

Every organ in the body is capable of performing just so much work, and no more, and it may be extended over a longer or shorter period; thus the habitual masturbator, with his constant practice of self-polution, the libertine, with his promiscuous venery, or the married man whose vows are but license to commit marital excesses, may, and often do, boast of their prowess, but in their excesses they are only drawing upon the future, and taking out of themselves forces they never will regain. Add to these conditions mental or physical overstrain, to which the present generation is so much subjected, and we have *all* the causes necessary to produce the most intractable form of sexual weakness.

The use, or rather abuse of stimulants and narcotics, is a fruitful source of sexual derangements. Every one knows that the intoxicated may and does have lascivious thoughts, but his power generally ends in his imagination. The withering effect of opiates upon the brain and spinal cord have ceased to be matters of speculation, and the alarming increase in the number of victims of this drug needs no argument to show its influence in producing the unfortunate condition under consideration. It perverts and destroys all the functions of the body. The use of chloral, cocaine and the coal tar preparations all tend to the same result, and while some of them, notably cocaine, may temporarily excite the sexual powers their continued use in the end brings exhaustion and with it sexual weakness and decay, for whatever produces extreme modifications of animal function will in the end bring about structural degeneration.

Heredity cuts no important figure in the induction of these conditions. Some of you may doubt this, but I do not. The heredity of sexual depravity is not denied, then why its weaknesses? If man can transmit his mental, moral and physical traits to his offspring, why not a weakness in his vital organism? It is a well established fact that injurious diseases or deep seated impressions on the central nervous system may be transmitted downward to the offspring, and I take it that few greater impressions are made on a man's mind than by the unhappy condition when in the very prime of life he discovers his manhood failing. As life organization and form descend from generation to generation, so may the diseases and infirmaties, and in the language of Benjamin W. Richardson, "The knowledge of such descent should increase our responsibility to the unborn. What evil I inflct on myself, what injury I inflict on others, may be transmitted to those yet unborn." Thus weakened nervous systems from any cause are liable to not only induce the conditions we are considering, but to be transmitted to the offspring also.

The next cause to which I shall allude is that of maternal impressions, and while some of you may not agree with me in the premises I assume, I hope to place the matter before you in such light as to at least merit attentive consideration. In "looking over the inhabitants of our own little city I have been profoundly impressed with the number of families who have no children, one or both sterile, going along the pathway of life hand in hand with no future in store but death and the grave. These people know none of the parental cares, joys, hopes or realizations. Their lives are without worldly stimuli, and their names without possibilities for the future. Some of them are but reaping what they sowed in their youth, while others go down by the waters of Marah and drink of the bitter cup provided by a wrecked or misguided ancestry, for every one of them willfully violating the provisions of nature, defying the laws of physiology or prostituting marriage rights in preventing the heaven ordained duty of bringing forth offspring is in some manner diseased or incapacitated, and the number thus situated is on the increase. Large as is the number of families who are childless they are much less numerous than that other class, able to bear children where the offspring is limited to the smallest possible number, one or at most two children, and it is this class that gives our profession so much trouble about means to prevent conception or gestation. Now, if one of these couples approach the marriage bed with no object in view but the gratification of an animal passion, all the time fearing and trembling lest conception should take place, if they should spend months or years planning against and opposing nature's legitimate results and finally in an unguarded moment or through some mishap "get caught," and on account of the failure of numerous and persistently tried means be forced to carry the unwelcome "child of an accident" to its full time and bring it into existence, need we be surprised if it should fail to have normal procreative powers? Children thus born may show no outward sign of sexual weakness or imperfection, but the mischief is there, and at an inopportune or premature time the latent wrong will become a developed fact, blasting hopes and sometimes happiness and homes. This picture is not overdrawn, and if you will eliminate from your cases of sexual weakness those directly traceable to causes produced by the individuals themselves, you will find a large majority of the remainder in persons descended from parents having small families.

Believing this as I do, I regard the prevention of child-bearing as one of the curses of this country, and I contend that to it is ascribable much of the increase of sexual weakness not alone among males but females as well, hence I am firmly impressed with the belief that "heredity and maternal impressions" play no unimportant role in the production of moral and physical decay of the human race.

Using means, by the male, to prevent conception is another source of sexual weakness, for whenever we prevent the natural processes of nature we weaken or destroy her powers, and I wish to call particular attention to plans used. First, wearing condoms or protectors, an invention of the French, who are said to be the most immoral and lascivious people in the pale of civilization, their use prevents direct contact of the parts, deprives them of their natural warmth and moisture, inhibits delicate nervous sensations, and robs the act of all its refined pleasures, their continued use ultimately obtunding the nerves of sensation and developing a species of sexual bashfulness or neurasthenia. The man is now sexually weak and liable to transmit the same unfortunate condition to his offspring should he accidentally have any.

The next practice to which I wish to refer is that of withdrawing from sexual congress as the act of emission is about to take place, an invention of the devil. It requires no arguments to show physiologists the evil results of this practice upon both mind and body. It is the worst in its effects of all means to prevent conception, and the man who practices it will sooner or later become a moral and physical wreck, and I wish to digress a little to say that the woman who requires either of these practices from her husband need not expect to long retain his affection or his fidelity unless he is mentally weak and badly henpecked, and if she does it will be a notable exception in the wide field of wrong doing around her. As one of our authors says in regard to raising and caring for children, "A woman who can not or will not forego the ephemeral pleasures and pastimes of society should eke out her life as a barren spinster."

It is not the premise of this paper to discuss symptoms or treatment, but to point out the causes of this growing evil to the human race in the prevention of which the church and medical profession are alike interested. To us as physicians is ascribed the duty of protecting and perfecting physical man, thereby perpetuating the race and giving stability and greatness to our common country. Knowing our duty we should not shrink from performing it fearlessly, faithfully and well, and then let us hope for happier days to come when under moral influences these maladies will practically cease to exist and the nosology of diseases be changed to strike from their list phenomena now too truly registered but then unknown except by the records of the past.

